# Rescue *in vitro* maturation of germinal vesicle oocytes after ovarian stimulation: the importance of the culture media

**DOI:** 10.1093/humrep/deaf099

**Published:** 2025-05-30

**Authors:** Nuria Soler, Danilo Cimadomo, Laura Escrich, Noelia Grau, Arancha Galán, Pilar Alamá, María José de los Santos, Laura Rienzi, María José Escribá

**Affiliations:** IVIRMA Global Research Alliance, IVI Valencia, Spain; IVIRMA Global Research Alliance, IVI Foundation—Fundación del IIS Hospital La Fe, Valencia, Spain; IVIRMA Global Research Alliance, Genera, Clinica Valle Giulia, Rome, Italy; IVIRMA Global Research Alliance, IVI Valencia, Spain; IVIRMA Global Research Alliance, IVI Valencia, Spain; IVIRMA Global Research Alliance, IVI Valencia, Spain; IVIRMA Global Research Alliance, IVI Foundation—Fundación del IIS Hospital La Fe, Valencia, Spain; IVIRMA Global Research Alliance, IVI Valencia, Spain; IVIRMA Global Research Alliance, IVI Foundation—Fundación del IIS Hospital La Fe, Valencia, Spain; IVIRMA Global Research Alliance, IVI Valencia, Spain; IVIRMA Global Research Alliance, IVI Foundation—Fundación del IIS Hospital La Fe, Valencia, Spain; IVIRMA Global Research Alliance, Genera, Clinica Valle Giulia, Rome, Italy; Department of Biomolecular Sciences, University of Urbino ‘Carlo Bo’, Urbino, Italy; IVIRMA Global Research Alliance, IVI Valencia, Spain; IVIRMA Global Research Alliance, IVI Foundation—Fundación del IIS Hospital La Fe, Valencia, Spain

**Keywords:** germinal vesicle oocytes, immature oocyte, rescue *in vitro* maturation, artificial oocyte activation, culture media

## Abstract

**STUDY QUESTION:**

What are the nuclear and initial developmental outcomes of rescue-IVM oocytes across multiple commercially available media?

**SUMMARY ANSWER:**

Of the 11 different media tested, Medium K provided the highest rescue rate in the shortest time and elicited the highest normal oocyte activation rate (NOAR).

**WHAT IS KNOWN ALREADY:**

Following ovarian stimulation, 10–15% of the oocytes are immature with about 10% states in prophase I, i.e. the germinal vesicle (GV) stage. A consensus is still needed whether to rescue these oocytes for clinical use or not, as reports of live births from rescued-metaphase-II (MII) oocytes continues to emerge. These oocytes may be valuable to poor prognosis patients when alternatives are not available and to oncofertility patients. Nonetheless, although rescue* in vitro* maturation (r-IVM) is experiencing a comeback, clear good practice recommendations regarding inherent protocols are lacking. Moreover, no commercially available culture media exist to support this potentially valuable rescue strategy.

**STUDY DESIGN, SIZE, DURATION:**

A prospective experimental study was conducted in a 1-year period at a private IVF center and entailing two consecutive phases. In study phase I, 1570 GV oocytes retrieved after ovarian stimulation from 490 young donors (maximum 4 per donor) were randomly cultured for 24 h in 11 commercially available culture media in a time lapse incubator. The two media eliciting the highest rescue rate in the shortest time were selected for study phase II. In this phase, 105 r-MII oocytes, obtained from 190 GVs rescued in the two chosen media, underwent artificial oocyte activation (AOA) and were further cultured in a time lapse incubator for 24 additional hours in Medium J until time of pronuclear fading (tPNf).

**PARTICIPANTS/MATERIALS, SETTING, METHODS:**

All donors (26.1 ± 3.8 years) underwent GnRH antagonist ovarian stimulation protocols with agonist trigger. The oocyte *in vivo* maturation rate was 80%. In the case of immature oocytes, two to four GV per woman were donated for research and cultured in an time lapse incubator. Time of GV breakdown (tGVBD) and time of the first polar body extrusion (t1PB) were annotated. AOA was conducted according to a previously published protocol. The primary outcome of study phase I was the rescue rate per cultured GV and the nuclear maturation dynamics in each medium studied. The primary outcome of study phase II was the NOAR of r-IVM oocytes in each selected medium. In this phase, time of pronuclear appearance and fading (tPNa and tPNf) and S-phase duration were also annotated.

**MAIN RESULTS AND THE ROLE OF CHANCE:**

The GVs cultured in 3 of the 11 tested media showed rescue rates ≥55%. However, the time to reach the r-MII in two of them, namely Medium G and Medium K, was significantly shorter (19.4 ± 0.2 h, 95%CI: 19.0–19.8 h). These two media were selected for study phase II. Following AOA, the NOAR obtained after rescue-IVM was significantly higher in the latter (n = 37/53, 70% vs. n = 21/52, 41%; *P* = 0.006, Power = 77%). No significant differences were observed in tPNa, tPNf or S-phase duration.

**LIMITATIONS, REASONS FOR CAUTION:**

The study was conducted in good-prognosis young oocyte donors and should be confirmed in poor-prognosis and/or advanced maternal age infertile women. Metaphase I (MI) immature oocytes were not included. AOA, which was used to assess initial oocyte competence (i.e. to resume meiosis and form a pronucleus), is useful for initial cytoplasmic competence but is an incomplete approach for the comprehensive assessment of cytoplasmic competence or further embryo development. In addition, tests such as microtubule and nuclear staining in r-MII to assess chromosome misalignment, organelle distribution and activity, non-invasive hyperspectral and AI oocyte analyses, more detailed morphodynamic assessments and, finally, analysis of meiotic segregation errors are mandatory to ensure the safety of r-MII oocytes prior to their potential clinical translation. Furthermore, due to the lack of knowledge regarding the qualitative and quantitative formulation of the commercially available culture media, to unravel the physiological mechanisms underlying the outcomes achieved is challenging.

**WIDER IMPLICATIONS OF THE FINDINGS:**

Among commercially available media not specifically designed for r-IVM, the media with glucose as main source of energy may show reduced rescue effectiveness. Conversely, the media with pyruvate as main source of energy, and combined with low lactate concentrations may elicit more favourable conditions to support both oocytes’ nuclear and initial cytoplasmic competence performance in GVs obtained after ovarian stimulation. This study provides first suggestions about which of the suboptimal systems would have the least detrimental effect on oocyte competence, thereby setting the stage for future appraisals for most effective rescue-IVM protocols.

**STUDY FUNDING/COMPETING INTEREST(S):**

This study was supported by the Instituto de Salud Carlos III, granted with the European Union (PI22/00924); the ‘Agencia Valenciana de la Innovación’ under the ‘Consolidación de la cadena de valor’ program of 2025 (INNCAD/2024/159) that is co-founded by the European Union through the ‘Programa Operativo FEDER’ de la Comunitat Valenciana 2021–2027, and by IVIRMA Valencia. The authors report no conflicts of interest related with the content of this manuscript.

**TRIAL REGISTRATION NUMBER:**

N/A.

## Introduction

Following ovarian stimulation, about 85% of the retrieved oocytes are at the metaphase-II (MII) stage and suitable for reproductive purposes. The other 15% are immature at either the germinal vesicle (GV, 11%) or metaphase I stages (MI, 4%) and, therefore, usually discarded. However, when the number of immature oocytes retrieved exceeds this proportion, the IVF cycle could be compromised. The possibility of increasing the number of MII oocytes could be achieved by an approach known as rescue IVM, a technique that allows GnRH-a/hCG-triggered cumulus-free immature oocytes to progress through meiosis *in vitro* (De Vos *et al.*, 2016, 2021), which is a different technological approach from conventional IVM, where harvested cumulus-oocyte complexes (COCs) are obtained from either mildly or non-stimulated ovaries without ovulation triggering (De Vos *et al.*, 2016, 2021). Despite the skepticism towards GV oocytes’ reproductive potential, some authors have rescued them for the clinical use and reported healthy live births (Veeck *et al.*, 1983; Nagy *et al.*, 1996; Nogueira *et al.*, 2000; Reichman *et al.*, 2010; Alcoba *et al.*, 2015; Lee *et al.*, 2016; Escrich *et al.*, 2018; Faramarzi *et al.*, 2018; Qin *et al.*, 2022). Based on this evidence, the Istanbul consensus working group recently updated their good practice recommendations regarding immature oocytes, suggesting that in ‘poor prognosis patients’ their use can be considered (a statement still subject to peer-review) (Coticchio *et al.*, 2025). Moreover, these oocytes may be valuable to oncofertility patients, even in association to typical treatment strategies adopted in these women (Segers *et al.*, 2015; De Vos *et al.*, 2016; Cadenas *et al.*, 2023; Nogueira *et al.*, 2023). This opens a window to re-start investigating the reproductive potential of rescued oocytes from immature eggs.

The effectiveness of the GV rescue depends—amongst other factors—on the culture medium used (Chian and Tan, 2002; Roberts *et al.*, 2002; Fesahat *et al.*, 2017), which should allow immature GV oocytes to progress to the MII stage, at a time suitable for clinical use (Escrich *et al.*, 2012). Most of the IVM media described to date involve harvesting cumulus-oocyte complexes (COCs) retrieved from either mildly or non-stimulated ovaries (Cha *et al.*, 1991; Trounson *et al.*, 1994, 2001; Chian *et al.*, 2000; Child *et al.*, 2001; Lin *et al.*, 2003; Le Du *et al.*, 2005; Soderstrom-Anttila *et al.*, 2005; Filali *et al.*, 2008; Chian and Cao, 2014), a scenario completely different from rescue-IVM of GV oocytes. Firstly, GV oocytes are retrieved from stimulated ovaries and after ovulation trigger by either hCG, a GnRH agonist or a combination of the two. Secondly, GV oocytes must complete meiosis in the absence of their companion gap junctional coupled cumulus cells, as they are typically removed to assess oocyte maturation state before ICSI or cryopreservation. As no specific culture medium has been developed so far to support nuclear and cytoplasmic maturation of cumulus-free immature oocytes retrieved after ovarian stimulation (i.e. rescue-IVM medium), we decided to test different complex culture media with or without supplements. The media tested and reported in the literature across the years are listed and described in Table 1: B2 medium (Janssenswillen *et al.*, 1995; Nogueira *et al.*, 2000; Torre *et al.*, 2006), HTF medium (human tubal fluid; Edirisinghe *et al.*, 1997; Farhi *et al.*, 1997; Chen *et al.*, 2000; Escrich *et al.*, 2011; Lee *et al.*, 2016), M-199 medium (Goud *et al.*, 1998; Vanhoutte *et al.*, 2007; Li *et al.*, 2019), Earle’s medium (Haberle *et al.*, 1999), TLP medium (Kim *et al.*, 2000), TCM-199 medium (Chian and Tan, 2002; Roberts *et al.*, 2002; Combelles *et al.*, 2005), MEME medium (Roberts *et al.*, 2002), Ham’s F10 medium (Fesahat *et al.*, 2017; Chatroudi *et al.*, 2019), and commercialized media for standard IVM like SAGE (CooperSurgical, CT, USA) IVM medium (Reichman *et al.*, 2010; Fesahat *et al.*, 2017; Kasapi *et al.*, 2017), and MediCult (CooperSurgical) IVM System (Al-Khtib *et al.*, 2011; Virant-Klun *et al.*, 2018). Of note, most of the studies supplemented the rescue medium with hormones (Reichman *et al.*, 2010; Fesahat *et al.*, 2017; Faramarzi *et al.*, 2018; Virant-Klun *et al.*, 2018; Chatroudi *et al.*, 2019), proteins (Nogueira *et al.*, 2000; Ben-Ami *et al.*, 2011; Lee *et al.*, 2016), serum (Edirisinghe *et al.*, 1997; Farhi *et al.*, 1997; Haberle *et al.*, 1999; Chen *et al.*, 2000; Nogueira *et al.*, 2000; Ben-Ami *et al.*, 2011; Alcoba *et al.*, 2015; Lee *et al.*, 2016), human follicular fluid (Kim *et al.*, 2000), Vero (Janssenswillen *et al.*, 1995; Nagy *et al.*, 1996; Farhi *et al.*, 1997; Nogueira *et al.*, 2000; Virant-Klun *et al.*, 2018; Chatroudi *et al.*, 2019; Esbert *et al.*, 2024) or sperm cells (Farhi *et al.*, 1997), or combining many of them also with amino acids, antibiotics, and/or salts (Farhi *et al.*, 1997; Goud *et al.*, 1998; Haberle *et al.*, 1999; Kim *et al.*, 2000; Chian and Tan, 2002; Combelles *et al.*, 2005; Vanhoutte *et al.*, 2007; Liu *et al.*, 2010; Al-Khtib *et al.*, 2011; Fesahat *et al.*, 2017; Kasapi *et al.*, 2017; Virant-Klun *et al.*, 2018; Chatroudi *et al.*, 2019; Li *et al.*, 2019). Recently, new strategies have been proposed to improve GV rescue performance. While co-culture with cumulus cells did not seem to improve the yield (Gordon *et al.*, 2023; Esbert *et al.*, 2024), the addition of various antioxidants, such as melatonin (Wei *et al.*, 2013; Zhu *et al.*, 2022), coenzyme Q10 (Ma *et al.*, 2020; Al-Zubaidi *et al.*, 2021), and resveratrol (Liu *et al.*, 2018) has shown promising results. Since commercial media with defined compositions, but undisclosed formulation, are commonly used in IVF laboratories to support gamete, early cleavages and post-transcriptional development to blastocysts, some of these media have also been used in rescue-IVM protocols, including G-10 medium (Vitrolife, Sweden) (Ben-Ami *et al.*, 2011), ECM medium (Early Cleavage Medium, Fujifilm Irvine Scientific, CA, USA) (Alcoba *et al.*, 2015), cleavage medium (Cook Medical, IN, USA) (Fesahat *et al.*, 2017), CCM medium (Vitrolife) (Escriba *et al.*, 2012; Escrich *et al.*, 2018), blastocyst medium (Cook Medical) (Fesahat *et al.*, 2017), and G2 medium (Vitrolife) (Faramarzi *et al.*, 2018; Esbert *et al.*, 2024).

**Table 1.** Summary of studies about rescue-IVM of fresh denuded germinal vesicle (GV) oocytes retrieved after ovarian stimulation and subsequent cytoplasmic competence assessed by fertilization after ICSI or parthenogenote production after artificial oocyte activation (AOA). Data were clustered according to the culture medium used.

	Supplements	Rescue rate	Fertilization/activation rate
**B2 Menezo (BioMerieux) medium**			
Janssenswillen *et al.* (1995)	—	Within 24 h: **20.9%** Within 30–36 h: **37.3%**	—
Janssenswillen *et al.* (1995)	Vero cells	Within 24 h: **47.6%** Within 30–36 h: **82.5%**	
Nagy *et al.* (1996)	Vero cells	Within 24 h: **64.3%**	**77.8%**
Nogueira *et al.* (2000)	HSA	Within 30–36 h: **67.8%**	**70.3%**
Nogueira *et al.* (2000)	Vero cells	Within 30–36 h: **74.5%**	
Torre *et al.* (2006)	Upgraded INRA Medium	Within 24 h: **43.1%** Within 44–48 h: **52%**	
**Human tubal fluid (HTF) medium**			
Edirisinghe *et al.* (1997)	Human Serum	Within 24 h: **100%**	**66.7%**
Farhi *et al.* (1997)	SSS (serum substitute supplement/synthetic serum substitute)	Within 24 h: **10%**	**50%**
Chen *et al.* (2000)	Patient’s/Maternal serum	Within 24 h: **27.4%** Within 44–48 h: **81.4%**	**58.5%**
Escrich *et al.* (2011)	—	Within 24 h: **55–66.4%** Within 44–48 h: **74.8%**	**22.4%**
Lee *et al.* (2016)	HSA (human serum albumin)	Within 24 h: **30.4%** Within 44–48 h: **67.8%**	**62.9%**
**Earle’s medium**			
Haberle *et al.* (1999)	Patient’s/maternal serum	Within 72 h: **73.8%**	**34.5%**
Haberle *et al.* (1999)	Cumulus Cells	Within 72 h: **64.9%**	**43.5%**
Roberts *et al.* (2002)		Within 24 h: **55%** Within 44–48 h: **74%**	
**M-199 WITH Earle’s salts**			
Combelles *et al.* (2005)	FSH, hCG, E_2_, HSA, glutamine, sodium pyruvate, penicillin, streptomycin	Within 24 h: **43.1%**	
Vanhoutte *et al.* (2007)	FSH, hCG, E_2_, HSA, glutamine, sodium pyruvate, penicillin, streptomycin	Within 24 h: **51%** Within 44–48 h: **72%**	
**TLP medium**			
Kim *et al.* (2000)	Glutamine, taurine, Earle’s, human follicular fluid, penicillin	Within 44–48 h: **65.0%**	**51.8%**
**TCM199**			
Goud *et al.* (1998)	FSH, hCG, E_2_, HSA, sodium pyruvate, penicillin, streptomycin	Within 24 h: **14.3%** Within 30–36 h: **33.9%**	53.8%
Goud *et al.* (1998)	FSH, hCG, E_2_, HSA, BSA, IGF, sodium pyruvate, penicillin, streptomycin	Within 24 h: **25%** Within 30–36 h: **64.3%**	**72.7%**
Chian and Tan (2002)	FSH, hCG, E_2_, SSS, sodium pyruvate, penicillin, streptomycin	Within 24 h: **34%** Within 44–48 h: **55.7%**	**78%**
Roberts *et al.* (2002)		Within 24 h: **22.8%** Within 44–48 h: **69%**	
Liu *et al.* (2010)	FSH, hCG, E_2_, EGF, FBS, sodium pyruvate, penicillin, streptomycin	Within 72 h: **63.6%**	
Li *et al.* (2019)	FSH, hCG, EGF, HSA, sodium pyruvate	Within 24 h: **35%**	**60.3%**
Li *et al.* (2019)	FSH, hCG, GH, EGF, HSA, sodium pyruvate	Within 24 h: **27.0%**	**73.1%**
**HAM’S F10**			
Chatroudi *et al.* (2019)	FSH, LH	Within 24 h: **63%**	**46.2%**
Chatroudi *et al.* (2019)	FSH, LH, cumulus cells	Within 24 h: **50%**	**76.5%**
Chatroudi *et al.* (2019)	FSH, LH, GDF-9	Within 24 h: **59.1%**	**46.2–75.5%**
Chatroudi *et al.* (2019)	FSH, LH, GDF-9, cumulus cells	Within 24 h: **57.6%**	**46.2–75.5%**
Fesahat *et al.* (2017)	HMG, FSH, LH, human follicular fluid	Within 24 h: **38%**	**52.7%**
**Own designed IVM medium**			
Chian and Tan (2002)	FSH, LH, E_2_, SSS	Within 24 h: **50.5%** Within 44–48 h: **75.7%**	**86.4%**
**IVM medium (MediCult IVM)**			
Al-Khtib *et al.* (2011)	FSH, hCG, patient’s/maternal serum	Within 30–36 h: **70.8%**	
Virant-Klun *et al.* (2018)	FSH, hCG	Within 44–48 h: **62.1%**	
Virant-Klun *et al.* (2018)	FSH, hCG, cumulus cells	Within 44–48 h: **77.2%**	
**Maturation medium (Sage, Copper Surgical)**			
Kasapi *et al.* (2017)	FSH, LH, SSS	Within 24 h: **67%** Within 24 h: **81.1%**	
Fesahat *et al.* (2017)	HMG	Within 24 h: **53%** Within 44–48 h: **68%**	**54.7%**
Reichman *et al.* (2010)	FSH, LH	Within 30–36 h: **35.1%**	**60%**
**Cleavage medium**			
Fesahat *et al.* (2017)	HMG	Within 24 h: **43%** Within 44–48 h: **53%**	**56.6%**
Alcoba *et al.* (2015)	SSS	Within 24 h: **58.5%**	**62.8%**
**G-1O (Vitrolife)**			
Ben-Ami *et al.* (2011)	HSA	Within 24 h: **36.5%**	**73.6%**
Ben-Ami *et al.* (2011)	Amphiregulin, Epiregulin, HSA	Within 24 h: **75.5%**	**71.8%**
**CCM (Vitrolife)**			
Escrich *et al.* (2012)	—	Within 24 h: **70%**	53.4%*
Escrich *et al.* (2018)	—	Within 24 h: **69.8%**	**64.2%**
**Blastocyst medium**			
Fesahat *et al.* (2017)	HMG	Within 24 h: **70%** Within 44–48 h: **78%**	**69%**
**G2 (Vitrolife)**			
Faramarzi *et al.* (2018)	HMG	Within 24 h: **66.3%**	**53.0%**

IVM,* in vitro* maturation.

When referring to culture duration information and oocyte progression to the MII stage (i.e. the rescue rate); the longer the culture (up to 30 h or even 48 h), the higher the rescue rates with both supplemented (Goud *et al.*, 1998; Chen *et al.*, 2000; Chian and Tan, 2002; Vanhoutte *et al.*, 2007; Lee *et al.*, 2016; Fesahat *et al.*, 2017; Kasapi *et al.*, 2017) and non-supplemented media (Janssenswillen *et al.*, 1995; Roberts *et al.*, 2002; Torre *et al.*, 2006; Escrich *et al.*, 2012). However, with respect to the time required for immature GV oocytes to resume meiosis and complete the first division, Escrich *et al.* (2012) defined two subpopulations, with oocytes that reached the MII stage within 24 h (18.4 ± 2.7 h) achieving higher activation rates than oocytes that reached this stage later. A dynamic critical also for developmental competence, as the same group reported in 2018 (Escrich *et al.*, 2018) that almost 70% of GVs rescued in CCM and reaching the MII stage within 24 h showed comparable *in vitro* and *in vivo* competence to their sibling MII oocytes.

In the present study, we aimed at investigating rescue rates (spontaneous nuclear meiotic progression) and initial cytoplasmic competence of rescued MII oocytes (r-MII) after GV rescue-IVM in eleven commercially available media.

## Materials and methods

**Figure 1.**
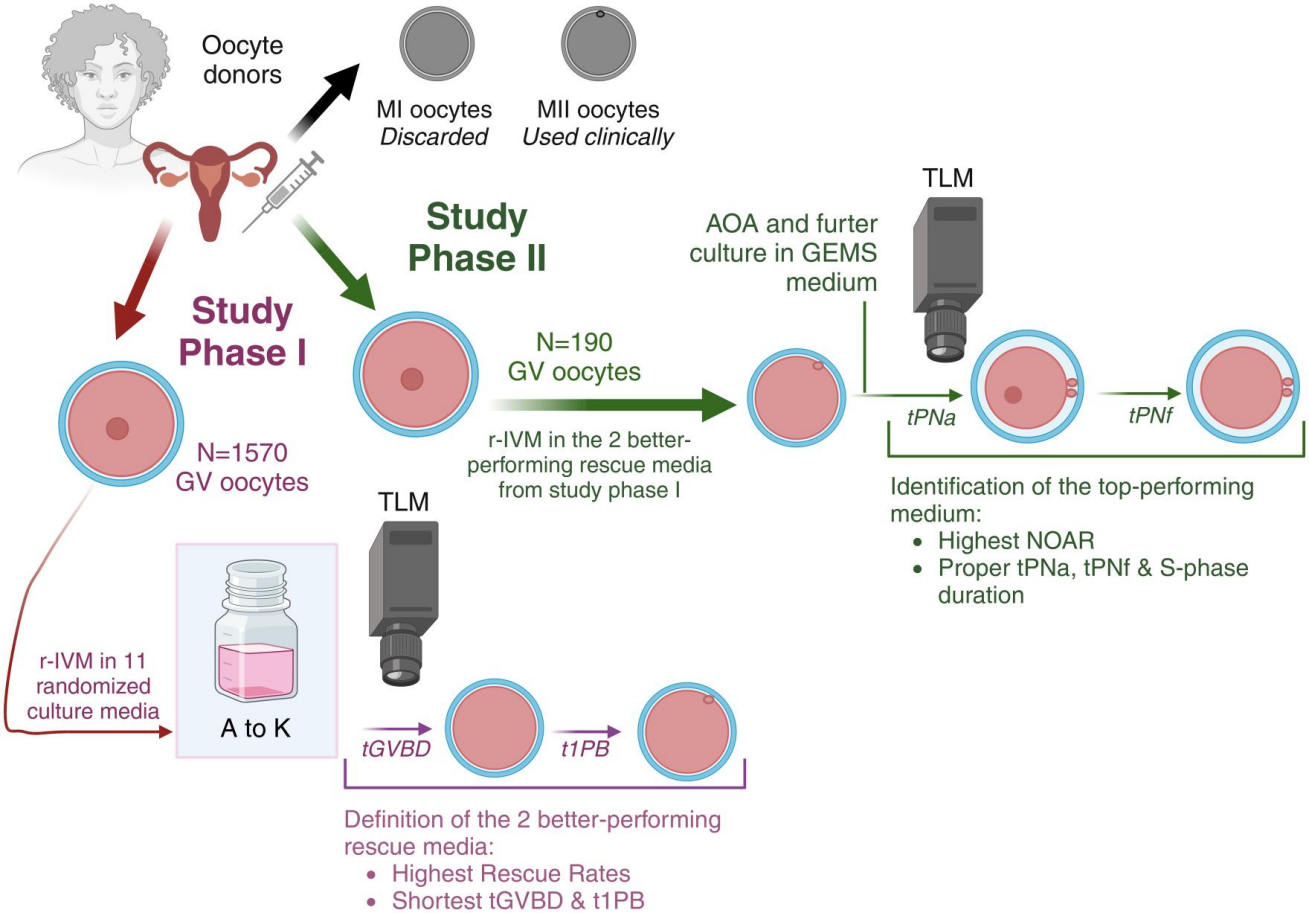
**Study workflow.** MII, metaphase II oocytes; MI, metaphase I oocytes; GV, germinal vesicle oocytes; r-IVM, rescue *in vitro* maturation; r-MII, rescued MII; tGVBD, time of GV breakdown; t1PB, time of first polar body extrusion; AOA, artificial oocyte activation; tPNf, time of pronuclear fading; NOAR, normal oocyte activation rate; tPNa, time of pronuclear appearance; S-phase, DNA-synthesis phases of the first cell cycle. Medium A–K, details are available in the Materials and Methods section. Created in BioRender. Rienzi, L. (2025) (https://BioRender.com/gjor27m).

The first phase of this study included 1570 immature GV oocytes, obtained from 490 young donors who underwent ovarian stimulation and oocyte retrieval for donation at IVI Valencia from February 2019 to March 2020. Overall, the GV oocytes were randomly cultured in 11 different culture media in time-lapse incubators to assess the rescue-IVM rate and nuclear kinetics according to the medium used. The two media with the highest rescue rates in the shortest culture time were then selected and compared for r-MII initial cytoplasmic competence after artificial oocyte activation (AOA) in a second phase of the study encompassing 190 additional GV oocytes (Fig. 1).

The study was approved by the Ethics Committee of the Instituto Universitario IVI (0703-E-402-ME and 2303-VLC-047-ME; Valencia, Spain) and written informed consent was obtained from all oocyte donors enrolled before retrieval.

### Experimental design

This prospective experimental study entailed two consecutive phases (Fig. 1).

In the first phase, we compared the rescue rates and the dynamics of oocyte nuclear maturation progression in 11 different commercially available culture media. We selected 11 commercial media to support the rescue-IVM. Four of these pertain to the commercial IVM MediCult system, without or with hormone supplementation (Media A–D). In particular, progesterone and estradiol were supplemented in Media B and D, as human oocytes do not have enough FSH and LH receptors, in turn potentially suffering from deficits in sex steroid hormone production. Media E includes the properties originally designed to support gametic physiology, while Media F represents a formulation that supports early developmental cleavages. Media G and H are selected as representatives of those that support blastocyst development; and finally, Media I–K are one-step media recently introduced into IVF laboratories to support the full spectrum of embryonic development. These media were: LAG medium of the MediCult IVM system [Medium A]; H-LAG (LAG medium of the MediCult IVM system supplemented with 10.3 nmol/l progesterone, 405.7 pmol/l estradiol [concentrations defined in house—data not shown] and 10% (v/v) HSA) [Medium B]; A-MIV (MIV medium of the MediCult IVM system supplemented with 10% (v/v) HSA) [Medium C]; H-MIV (MIV medium of the MediCult IVM system supplemented with 10. 3 nmol/l progesterone, 405.7 pmol/l estradiol [concentrations defined in house—data not shown] and 10% (v/v) HSA) [Medium D]; Sydney IVF fertilisation medium (Cook Medical) [Medium E]; Sydney IVF cleavage medium (Cook Medical) [Medium F]; G-2 PLUS (Vitrolife) [Medium G]; CSCM (Fujifilm Irvine Scientific) [Medium H]; GLOBAL TOTAL (Life Global, Cooper Surgical) [Medium I]; GEMS (Geri Medium, Genea BioMedx, Australia) [Medium J]; and CSCM-NXC (Fujifilm Irvine Scientific) [Medium K]. Supplementary Table S1 summarizes the presence/absence of major energy sources (glucose, lactate, and pyruvate) and essential and non-essential amino acids in the 11 media. The information was retrieved from Morbeck *et al.* (2014) and Zagers *et al.* (2025).

In the second phase, the two media with the highest rescue rates in the shortest culture time were selected. Only r-MII oocytes matured within 24 h were assessed for initial cytoplasmic competence by AOA. Normal oocyte activation rates (NOAR) and first cell-cycle morphokinetics of parthenogenotes were compared between the two selected media.

### Donors’ characteristics

All women included who donated immature eggs for this study were enrolled at the private IVF center IVI Valencia. They underwent ovarian stimulation between February 2019 and March 2020 and signed the informed consent to the study. The oocyte donors’ age was 18–35 years, they had regular menstrual cycles, no Mendelian or chromosomal conditions, a body mass index of 18–28 kg/m^2^, normal karyotype, no sexually transmitted diseases, and a normal ovarian reserve characterized by AMH levels >1.5 ng/ml or antral follicle counts >12 follicles in both the ovaries at the beginning of the cycle; the use of GV oocytes from donor women eliminates any potential infertility factors related to the female partner, ensuring that the results are not confounded by maternal age or other reproductive issues. This approach allows for a more accurate assessment of the rescue-IVM culture media used, while demographic and reproductive parameters help maintain the homogeneity of the donor group.

### Ovarian stimulation protocols

Oocyte donors underwent ovarian stimulation with recombinant follicle-stimulating hormone (rFSH) and/or human menopausal gonadotropin (hMG) and 10 mg of medroxyprogesterone acetate (MPA) daily, following the routine clinical practice at IVI Valencia, as described elsewhere (Giles *et al.*, 2021). Briefly, throughout ovarian stimulation until ovulation induction, daily FSH and/or hMG were administered in combination with a single daily oral dose of MPA. Ovulation induction was triggered with a single subcutaneous bolus of 0.2 mg of triptorelin, when 3 or more follicles ≥18 mm were confirmed by transvaginal ultrasound (Melo *et al.*, 2009). Follicular aspiration was performed under sedation and transvaginal ultrasound guidance, following 36 h after ovulation induction.

### Oocyte retrieval and GV oocytes collection

Following follicle aspiration, COCs were retrieved, washed, and incubated in fertilization medium (Cook Medical) at 37°C, 6%CO_2_ and 5%O_2_ for 2 h before denudation. Cumulus cells were removed by gentle pipetting after a brief incubation in hyaluronidase (40 IU/ml; Sage IVF). Cumulus-free oocytes were classified as MII or immature; the latter included those oocytes with no polar body (PB) or nuclear structure (i.e. metaphase I oocytes, MI) or GV oocytes, featured by a GV structure in the cytoplasm and no PB in the perivitelline space.

GV oocytes were identified and randomly assigned to different media. A maximum of four GV oocytes per donor and oocyte retrieval were included to minimize any potential cycle-related bias.

### GV rescue-IVM

Every day, one or two Embryoscope slides (12 or 24 individualized wells) were prepared by an embryologist not involved in the allocation phase. Each slide was fully filled with a commercial medium according to the randomization list and experimental phase. The day after, if two or more GVs were identified from each donor, they were asked to participate in the study. Each donor could contribute up to 4 GV oocytes. After obtaining consent from them, GVs were anonymized and cultured individually, in the order they appeared, in an Embryoscope slide containing the medium that was blinded to the embryologists making the allocation. The same process was followed until study completion.

After allocation, GV oocytes were cultured in 25 µl of medium in an Embryoscope (Vitrolife) incubator (at 37°C, 6% CO_2_ and 5% O_2_) for 24 h. For all media studied (Media A–K), the rescue rate was assessed as the percentage of cultured GVs that progressed to the MII stage within the first 24 h of culture (r-MII). In addition, the dynamics of meiotic progression was described by the time of germinal vesicle breakdown (tGVBD) and the time of first PB extrusion (t1PB), with oocyte denudation considered as time zero. These two time points outline the onset of MI and MII, respectively, and the difference between them quantifies the duration of MI arrest.

### Oocyte activation

Two hundred GV oocytes were randomly assigned to the two best rescue-IVM media. After retrospective re-evaluation, 10 GVs were excluded due to signs of atresia or degeneration, leaving 190 viable GV oocytes (97 GVs in the Medium G and 93 GVs in the Medium K). A total of 105 r-MII oocytes reached this stage across both media (52.5%), with 52 r-MII oocytes in Medium G and 53 r-MII oocytes in Medium K. These oocytes subsequently underwent AOA, following the protocol described by Escriba *et al.* (2016). Briefly, within 2–4 h from t1PB, oocytes were incubated in 8 µM A23187 (Sigma-Aldrich, MO, USA) for 5 min and then in 10 μg/ml puromycin (Sigma-Aldrich) for 5 h. After AOA, oocytes were cultured in a time-lapse incubator in single-step medium (GEMS, Genea BioMedx). After 16–20 h of culture, r-MII were assessed for the second PB extrusion and pronuclear number. Although several types of human oocyte activation response have been reported (Escrich *et al.*, 2011), only bio-constructs (i.e. parthenogenotes) that had extruded the second PB and had a single PN were considered for the calculation of the NOAR as a percentage of r-MII initially incubated in activation solutions. The precise timing of PN appearance and fading (tPNa and tPNf, respectively) was checked in terms of hours after calcimycin incubation. As an indirect variable, the duration of the S-phase was also calculated as the difference tPNf–tPNa. The development of parthenogenotes was not further evaluated.

### Statistical analysis

Categorical variables were expressed as number of cases (N) and prevalence (%) with 95% CI, where relevant, and compared using Fisher’s exact tests. Statistical significance was set at a *P*-value of 0.05.

Continuous variables, such as all timings, were presented as mean and standard deviation and tested for normality using the Kolmogorov–Smirnov test. Data were adjusted for normal distribution and analyzed by analysis of variance (ANOVA) according to the media used. In study phase II, *t*-test for independent samples was used. Statistical significance was set at a *P*-value of 0.05.

All statistical analyses were performed using SPSS version 29.0.2.0 (IBM, New York, USA). Post-hoc power analyses were conducted with G*Power.

## Results

The mean donor age was 26.1 ± 3.8 years with a mean BMI of 24.3 ± 3.6 kg/m^2^. They were stimulated for 10.1 ± 1.3 days with a mean total FSH dose of 1866.1 ± 739.8 IU, and a mean total hMG dose of 600 ± 1012.7 IU. A total of 21.2 ± 9.2 oocytes were retrieved per donor, of which 16.9 ± 7.7 were MII, giving a mean retrieval maturation of 80.1%. On average, 3.2 ± 0.5 GV oocytes were identified. A total of 1760 GV oocytes were included in the study (phase 1: 1570 GVs; phase 2: 190 GVs) and cultured *in vitro*.

**Figure 2.**
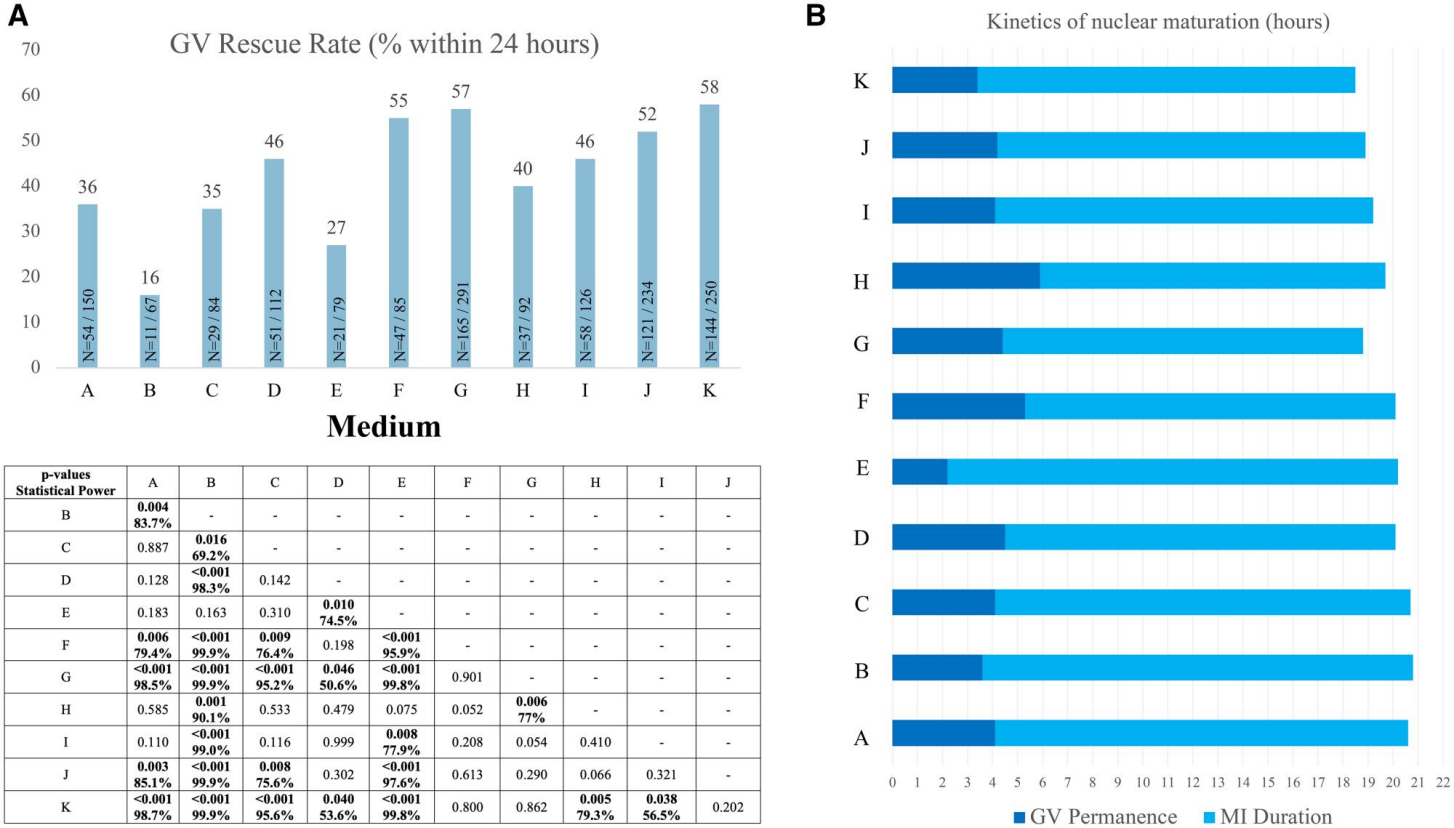
**Rescue rate and kinetics of the transition from the germinal vesicle (GV) to metaphase II (MII) stage among 11 commercially available culture media.** (**A**) GV rescue rates within 24 h of culture. The histogram shows the rates and the absolute numbers, while the matrix below shows the *P*-values of each comparison and the statistical power reached by all significant differences. (**B**) Nuclear dynamics of the GV oocytes that reached the MII stage within 24 h of culture, according to the different commercially available media tested. The deep blue bars represent the average time of permanence at the GV stage, in hours. The light blue bars represent the average time of permanence at the metaphase I (MI) stage in hours. The whole bars represent the average total time required for spontaneous completion of the first meiosis from the GV to the r-MII stage, outlined by the extrusion of the first polar body. Medium A–K, details are available in the Materials and Methods section.

In study’s phase 1, 738 out of 1570 GV oocytes reached the MII stage within 24 h of culture (rescue rate: 47%). After examining the rescue-IVM videos, we found significant differences in both the rescue rates and the kinetics of nuclear maturation according to the medium used. Regarding the rescue rate, our results showed that GVs cultured in media F, G, J, and K showed a rescue rate >50%. Rates higher than what was achieved in media D and I (about 46%), H (40%), A and C (about 35–36%), or B and E (<30%; Fig. 2A). Figure 2A highlights all the statistically significant differences for each comparison with the related statistical power.

The dynamics of nuclear maturation were examined in all 738 r-MII and compared according to the media tested (Fig. 2B). The GVs cultured in media G and K reached the MII stage at comparable times (19.4 ± 0.2 h, 95%CI: 19.0–19.8 h) and significantly earlier (*P* = 0.001) than those rescued in media A–D (22.4 ± 0.2 h, 95%CI: 22.0–22.8 h) or in media E, F, H, I, or J (mean t1PB: 20.4 ± 0.2 h, 95%CI: 20.0–20.8 h). Examination of specific nuclear maturation events regarding GVs rescued in media G and K showed significant differences in both the time of GVBD onset (4.4 ± 0.2 h vs 3.4 ± 0.2 h, *P* = 0.001) and the duration of the MI stage (14.6 ± 0.2 h vs 15.1 ± 0.1 h, *P* = 0.001). Analysis of the major events in the nuclear meiotic progression of r-MII oocytes within 24 h of culture showed that the differences were mainly due to either a longer permanence in the GV stage when r-MII had been rescued in media F or H (5.5 ± 0.3 h) or a longer permanence in the MI stage when cultured in media B or E (17.7 ± 0.4 h).

**Figure 3.**
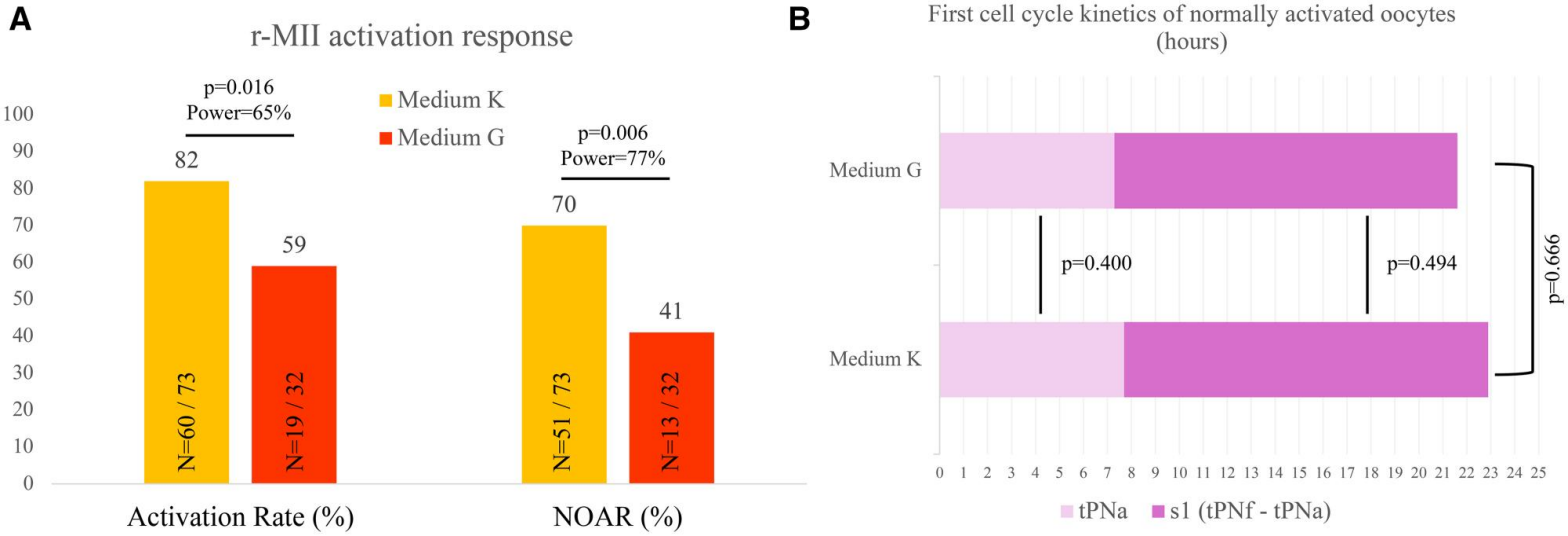
**Post-maturation effects of the culture media that yielded the highest rescue rates in the shortest timeframe.** Rescued MII oocytes’ activation rates (overall and normal oocyte activation rate [NOAR]) (**A**) and first cell-cycle kinetics of the parthenogenotes (**B**). tPNa, time of pronucleus appearance; tPNf, time of PN fading; s1, duration of the first cell cycle’s S-phase. Medium G (G-2 PLUS: Vitrolife) and Medium K (CSCM-NXC: Fujifilm Irvine Scientific).

After selecting G and K as the media with the highest rescue rates in the shortest time (57.1% in an average of 19.4 ± 0.2 h), we studied the response of r-MII oocytes to AOA. In Phase II, the previously observed rescue rates were confirmed (average 55.3%) and there were also no differences between media G and K (53.6% and 57% respectively; *P* = 0.7). A significantly higher percentage of r-MII rescued in medium K (n = 53) were activated and eventually showed a NOAR (82.2% and 69.9%, respectively) than those rescued in medium G (n = 52; 59.4% and 40.6%, respectively) (Fig. 3A). Irrespective of the rescue media used, bioconstructs (parthenogenotes) showed comparable morphokinetics throughout the first cell cycle, as shown by tPNa (Medium K: 7.7 ± 1.5 h vs Medium G: 7.3 ± 0.5 h; *P* = 0.400), tPNf (Medium K: 22.7 ± 6.2 h vs Medium G: 21.5 ± 0.3 h; *P* = 0.666) and S-phase length (s1) (Medium K: 15.2 ± 2.8 h vs Medium G: 14.3 ± 0.5 h; *P* = 0.494) (Fig. 3B).

## Discussion

The clinical use of rescued IVM oocytes in an IVF cycle is an old idea that has not been clinically established as the results are still poor, and more research is needed to improve the developmental competence of these oocytes, being the availability of suitable media a first crucial step. Rescue-IVM is currently being re-evaluated by the scientific community, as evidenced by the continuing reports of live births from r-MII oocytes and the recent update of the ESHRE-Alpha Istanbul consensus (Coticchio *et al.*, 2025). Indeed, although r-MII oocytes have been reported to be generally less competent than sibling MII oocytes, they can develop to the blastocyst stage at comparable rates and ultimately contribute to live birth rates per attempt. This strategy represents a potentially valuable resource, especially for women with low functional ovarian reserve (Nicholas *et al.*, 2023) or when the number of MII at retrieval was lower than expected by folliculometry (Escrich *et al.*, 2018; Yilmaz *et al.*, 2022), representing women with a compromised follicular output index (FOI) (van der Gaast *et al.*, 2006), referred to as MII oocytes. Furthermore, this approach could serve as a rescue strategy for completely or partially denuded oocytes, which are typically excluded from standard IVM protocols due to their lower developmental competence and altered transcriptomic profiles compared to cumulus-enclosed oocyte complexes (Luciano *et al.*, 2023; Pham *et al.*, 2024; Cava-Cami *et al.*, 2025). In addition, it may offer advantages for GV oocytes collected in conjunction with ovarian tissue cryopreservation protocols, expanding its potential applications in fertility preservation (Revel *et al.*, 2003; Huang *et al.*, 2008; Escriba *et al.*, 2012). Nonetheless, rescue-IVM is a time-consuming procedure whose cost-effectiveness and long-term follow-up remains to be evaluated. Therefore, its clinical implementation requires careful protocol validation, standardization and preclinical appraisal. In view of this, r-IVM could have a role in boosting the success rates of ART in low responders and in oncofertility, but whether r-IVM is worth the effort in other patients is questionable.

The aim of this study was to determine which of the suboptimal systems would have the least detrimental effect on oocyte competence, making them ready for use in the current clinical IVF routine. It is also the aim of this work to highlight the lack of standardization in the literature regarding media and, with the results presented, to provide a starting point for standardizing rescue IVM protocols for cumulus-free GV oocytes retrieved after ovarian stimulation.

### Biological factors and culture conditions

The ability of GV oocytes to progress to competent r-MII oocytes depends, among other factors, on patient-specific biological characteristics, variables that are beyond the scope of this study (for a review see (Capalbo *et al.*, 2021; Paonessa *et al.*, 2021; Innocenti *et al.*, 2022)). The competence of human r-MII oocytes can then be significantly influenced by the culture conditions, in particular by the culture medium (Chian and Tan, 2002) and the t1PB (Escrich *et al.*, 2012). Indeed, culture conditions can significantly impact on the ability of immature oocytes to complete meiosis *in vitro* (Roberts *et al.*, 2002; Sutton *et al.*, 2003) by acting upon gene expression, kinetics of cell cycle, and spindle/chromatin organization, in turn affecting subsequent embryonic development (Nogueira *et al.*, 2000). However, as immature GV oocytes represent a low percentage of retrieved oocytes (about 10%) (Janssenswillen *et al.*, 1995; Farhi *et al.*, 1997) and their reproductive potential is still under appraisal, there has been a limited commercial interest in developing culture media specifically designed to support the final stages of meiotic maturation in immature oocytes retrieved after ovarian stimulation. However, our results suggest that potential clinical applicability may be closer than expected if improvements in rescue IVM can increase the number of available MII oocytes, especially in cases where their number is compromised (Lee *et al.*, 2016) or does not match the expected number based on folliculometry (Escrich *et al.*, 2018). Furthermore, this approach could serve as a rescue strategy for completely or partially denuded oocytes, which are typically excluded from standard IVM protocols due to their lower developmental competence compared to cumulus-enclosed oocyte complexes. In addition, it may offer advantages for GV oocytes collected in conjunction with ovarian tissue cryopreservation protocols, expanding its potential applications in fertility preservation (Revel *et al.*, 2003; Huang *et al.*, 2008; Escriba *et al.*, 2012).

After a careful revision of the literature (summarized in Table 1), it was evident that most culture media used to date for rescue-IVM were either home-made or commercial media supplemented with hormones. Although these media were originally designed to mature entirely cumulus-enclosed oocytes, recovered from mild or unstimulated cycles without trigger, they led to rescuing immature oocytes in a setting encompassing cumulus-free gametes obtained after ovulation trigger (Ben-Ami *et al.*, 2011; Lee *et al.*, 2016). Some of these media were tested in our work, but they reached modest rescue rates within 24 h (about 40%; Media A and C). The practice of sex steroid supplementation then appeared even detrimental here, with Medium B reaching less than 20% rescue rate. Comparable low results (rescue rates below 30%) were achieved using medium E in this study, echoing the results published by other authors using media like the B2 Ménezo medium or TCM 199 (Janssenswillen *et al.*, 1995; Roberts *et al.*, 2002; Torre *et al.*, 2006) and non-supplemented HTF (Lee *et al.*, 2016). When testing media designed to support early preimplantation embryo development until the cleavage stage (Day 3), the rescue rates achieved were generally ≤40% (Ben-Ami *et al.*, 2011; Fesahat *et al.*, 2017). Instead, much better outcomes were achieved using media designed to support late preimplantation embryo development to blastocyst, with rescue rates as high as 70% (Escrich *et al.*, 2011; Faramarzi *et al.*, 2018). Indeed, also in the present study, media G, J, and K achieved rescue rates higher than 50%, and—along with media H and I whose rescue rates were 40% and 46%—they represent this category of culture media.

In general, all the 11 media investigated in the present study sustained meiotic progression of GV oocytes to MII, although to different extents. We hypothesized that the nature of the main energy source—namely the presence of either glucose and/or pyruvate (Morbeck *et al.*, 2014; Zagers *et al.*, 2025) and their reciprocal proportion—was at the root of this variability (Supplementary Table S1). In fact, despite glucose receptors being present in human oocytes, it seems that cumulus-free oocytes exhibit low or null glycolytic activity, attributed to reduced PFK activity (Purcell *et al.*, 2012; Wale and Gardner, 2012). Therefore, oocytes utilize pyruvate for energy metabolism through oxidative phosphorylation via the tricarboxylic acid (TCA) circuit and electron transfer, which serves as an important source of energy during meiotic maturation (Donahue and Stern, 1968; Johnson *et al.*, 2007). Normally, pyruvate is produced from the cumulus cells and transferred to the oocyte through the gap junctions. However, in the absence of cumulus cells, GV oocytes cannot utilize glucose to produce pyruvate (Harris *et al.*, 2007; Wilding *et al.*, 2009; Sutton-McDowall *et al.*, 2010). The direct uptake of pyruvate from the culture medium (Biggers *et al.*, 1967) and a metabolism based on the TCA cycle and electron transfer pathways produces lactate as a by-product which can either be released into the environment in a pro-gradient manner or converted to useful pyruvate with the consequent consumption of NADH and increase in intracellular oxidative levels. During oocyte *in vitro* progression to MII, lactate accumulates in the culture medium at the GVBD stage due to increased pyruvate uptake (Roberts *et al.*, 2002). Thus, in an extracellular environment that favors the release of the lactate by-product, as may be the case in the low lactate media, the metabolic pathway of pro-oxidative phosphorylation through the citric acid cycle is enhanced and the oxidative state mitigated (Kobanawa, 2022). The fine regulation of these metabolic pathways may explain the variability in the results showed by this study.

Concerning amino acids, the oocyte can also use them as energy sources or as biosynthetic precursor molecules, osmolytes, buffers of internal pH, antioxidants and chelators, especially for heavy metals. However, the oocyte is unable to uptake certain amino acids, including the non-coupling-dependent amino acid L-Alanine. Consequently, they depend on cumulus cells to supply these nutrients or may adapt by utilizing alternative metabolic pathways.

As the embryo amino acid requirement changes during preimplantation development, sequential culture systems involve a transition from media lacking or showing reduced concentration of essential amino acids to media with a high content of all amino acids, mostly essential in nature (Morbeck *et al.*, 2014) (Supplementary Table S1). Whether amino acid content and their concentration are related to the competence of the r-MII is beyond our scope. Nevertheless, this is an interesting area of investigation as the turnover of valine and alanine, and the content of glutamic acid, glutamine, arginine and isoleucine were all associated with the rescue rate of human (Hemmings *et al.*, 2013) and previously also bovine (Brison *et al.*, 2004) immature oocytes.

### Metabolism and meiotic progression

GV oocytes metabolism is characterized by active transcription and translation during the pre-ovulatory period (Ducreux *et al.*, 2023). However, transcription ceases upon meiotic resumption, leaving the oocyte and the early pre-embryo relying upon the pool of mRNAs and proteins accumulated in the ooplasm during oogenesis. In this regard, Roberts and colleagues (Roberts *et al.*, 2002) observed that the faster the oocytes progress through meiosis, the lower the pyruvate uptake and lactate production detected. As metabolism increases, both lactate and NAD+ production increase and accumulate in the culture medium in a reversible, near-equilibrium reaction. In mice, increased lactate in the medium was reported to decrease oocyte metabolism by reducing the oxidative use of pyruvate (Tarahomi *et al.*, 2019). This imbalance may affect gamete metabolism pathways, promoting pyruvate conversion by lactate dehydrogenase (LDH) or leading to lactate accumulation in the medium. CSCM-NXC (Medium K here), a low lactate medium, may favour the lactate to pyruvate (L/P) ratio and reduce reactive oxygen species (ROS) levels, thereby improving oocyte metabolic efficiency. The beneficial effect of reducing the oxidative stress is testified by the increased percentage of usable blastocysts in advanced maternal age women reported by Hammond and Morbeck (2020) and Kobanawa (2022) and here presented by the high rescue rates achieved and oocyte activation response displayed in CSCM-NXC (Medium K). Conversely, G-2 Plus medium (here Medium G), presents a high glucose and low pyruvate content and a high L/P ratio compared to other continuous culture media (Tarahomi *et al.*, 2019). This energy supply may force the oocyte to use alternative sources (namely amino acids), which in turn may stress the gamete and affect its cytoplasmic maturation (Tarahomi *et al.*, 2019), as suggested by the high rescue rates but low activation rates observed with this medium in this study. In this regard, though, meiosis II completion in maternal bio-constructs is insufficient to define oocyte competence. Therefore, the exploration of r-MII oocytes’ competence after rescue in CSCM-NXC medium now requires assessment of ROS levels in the oocytes, redox state (NAD^+^/NADH and NADP^+^/NADPH), oocyte metabolomics, analysis of spent culture media, chromosomal alignment and spindle morphology, and analysis of meiotic segregation errors. The current absence of these data must be considered a limitation. Besides, when dealing with commercially available media, it is challenging to outline a reliable physiological explanation to the outcomes achieved as the different molecular compositions and concentrations are not fully disclosed by the companies.

Research on human cumulus-free GV oocytes retrieved from stimulated cycles remains limited, and prospective studies on defined culture conditions for immature oocyte rescue are scarce. Recent prospective studies on rescue media supplementation have involved small sample sizes, with findings suggesting that co-culture with cumulus cells or the addition of rapamycin to the culture medium does not affect rescue rates (Esbert *et al.*, 2024). However, increased numbers of MII oocytes have been observed following supplementation with follicular fluid and cumulus-granulosa cell supernatant (Madkour *et al.*, 2018), as well as with melatonin (Wei *et al.*, 2013), CoQ10 (Ma *et al.*, 2020), GDF9-β (Mohsenzadeh *et al.*, 2022), and a synthetic tripeptide derived from human fertilin (Sallem *et al.*, 2022). These findings highlight a new paradigm for the needs of cumulus-free immature oocytes retrieved from stimulated cycles.

Finally, while doubts about the viability of rescued oocytes are valid, it is undeniable that this field of study is still in its early and challenging stages. This context highlights the urgent need to establish fundamental guidelines in protocols, as proposed in this work, with the goal of moving toward a clearer and more defined future. In this innovative and exploratory phase, while being cautious against adopting rescue-IVM as a ‘rescue-all’ strategy for universal application, it would also be unwise to dismiss cases where this technique could have a positive impact. The reality is that, just like any cohort of mature oocytes (MII), not all immature oocytes will necessarily be viable, nor will all be non-viable. In this regard, this work has a pioneering role in deepening our understanding and methods of identification, with the firm goal of paving the way for their effective and precise rescue, opening this perspective for specific populations of IVF patients.

## Conclusions

This study suggests that nuclear maturation and cytoplasmic competence must proceed together. While the t1PB extrusion is the main criterion to assess oocytes’ nuclear competence, the tGVBD and the duration of MI might also be relevant to predict their overall competence, partially explaining the differences in activation rates between different culture media. Further research is needed to fully understand the biological relevance of these observations and to optimize culture conditions for GV rescue-IVM. Apparently, the use of glucose as main source of energy may reduce rescue-IVM effectiveness, even after medium supplementation, whereas media based on low L/P may involve more favourable conditions to support both oocytes’ nuclear and cytoplasmic competence, at least in rescued GVs obtained after ovarian stimulation from good prognosis young women. Although our discussion has focused on the qualitative composition of pyruvate and lactate in the commercial media tested, no quantitative determinations could be made. Furthermore, it is possible that other significant differences in media composition—like amino acids as optional sources of energy, osmolarity, pH, hormonal supplements, or other additives of biological origin—may be responsible for the observations reported in this article.

In conclusion, in this work, we relied on commercially available culture media specifically designed for preimplantation embryo culture and addressed their suitability for GV rescue-IVM purpose. Unfortunately, the compositions and formulations of these media are not fully disclosed by the companies, thus inciting future studies to formulate more in-depth speculations of the physiology behind rescue-IVM efficiency. Additional studies shall also focus on poor prognosis and/or advanced maternal age women, include MI immature oocytes, and monitor the developmental, chromosomal and reproductive competence of r-MII oocytes. These data are mandatory to ensure the safety of r-MII oocytes prior to their ordinary clinical use in IVF.

## Supplementary Material

deaf099_Supplementary_Table_S1

## Data Availability

The data underlying this article are available in the article and in its online supplementary material. Images and videos are not publicly available.
