# Supplementary material for: Rescue in vitro maturation of germinal vesicle oocytes after ovarian stimulation: the importance of the culture media
Source: Hum Reprod. 2025 May 30;40(8):1504–15. doi: 10.1093/humrep/deaf099 (PMC12314148; doi:10.1093/humrep/deaf099)
Supplement: deaf099_Supplementary_Table_S1 [file deaf099_supplementary_table_s1.pdf]

Human Reproduction, 2025, 1–1
https://doi.org/10.1093/humrep/deaf099
Supplementary Data

Supplementary Table S1. Presence/absence of major energy sources (glucose, lactate, and pyruvate) and essential and non-essential amino acids in the eleven selected media tested for GV Rescue-IVM (Medium A–K).

	Medium A	Medium B	Medium C	Medium D	Medium E	Medium F	Medium G	Medium H	Medium I	Medium J	Medium K
**Main energy sources**											
Glucose	YES	YES	YES	YES	YES	NO	YES	NO	N/A	NO	NO
Lactate	NO	NO	NO	NO	YES	Low	YES	YES	N/A	YES	NO
Pyruvate	NO	NO	NO	NO	YES	YES	Low	YES	N/A	YES	YES
**Essential amino acids**											
Histidine	NO	NO	YES	YES	NO	NO	YES	YES	N/A	YES	YES
Isoleucine	NO	NO	YES	YES	NO	NO	NO	NO	N/A	NO	NO
Leucine	NO	NO	YES	YES	NO	NO	YES	YES	N/A	YES	YES
Lysine	NO	NO	YES	YES	NO	NO	YES	YES	N/A	YES	YES
Methionine	NO	NO	YES	YES	NO	NO	YES	YES	N/A	YES	YES
Phenylalanine	NO	NO	YES	YES	NO	NO	YES	YES	N/A	YES	YES
Threonine	NO	NO	YES	YES	NO	NO	YES	YES	N/A	YES	High
Tryptophan	NO	NO	YES	YES	NO	NO	YES	YES	N/A	YES	YES
Valine	NO	NO	YES	YES	NO	NO	YES	YES	N/A	YES	YES
**Non-essential amino acids**											
Alanine	NO	NO	YES	YES	YES	YES	YES	YES	N/A	NO	YES
Arginine	NO	NO	YES	YES	NO	Low	YES	YES	N/A	YES	YES
Asparagine	NO	NO	YES	YES	YES	YES	YES	YES	N/A	YES	YES
Aspartic Acid	NO	NO	YES	YES	YES	YES	YES	YES	N/A	YES	YES
Glutamine	NO	NO	YES	YES	YES	YES	YES	YES	N/A	NO	High
Glutamic Acid	NO	NO	YES	YES	YES	YES	YES	YES	N/A	YES	YES
Glycine	NO	NO	YES	YES	YES	YES	YES	YES	N/A	YES	YES
Proline	NO	NO	YES	YES	YES	YES	YES	YES	N/A	YES	YES
Serine	NO	NO	YES	YES	YES	YES	YES	YES	N/A	YES	YES
Tyrosine	NO	NO	YES	YES	NO	Low	YES	YES	N/A	YES	YES

N/A: not available. Based on Morbeck *et al.* (2014) and Zagers *et al.* (2025).

© The Author(s) 2025. Published by Oxford University Press on behalf of European Society of Human Reproduction and Embryology.
This is an Open Access article distributed under the terms of the Creative Commons Attribution License (https://creativecommons.org/licenses/by/4.0/), which permits unrestricted reuse, distribution, and reproduction in any medium, provided the original work is properly cited.
Human Reproduction, 2025, 00, 1–13
https://doi.org/10.1093/humrep/deaf099
Original Article
